# A Simulation Study on Sieving as a Powder Deposition Method in Powder Bed Fusion Processes

**DOI:** 10.3390/ma17143382

**Published:** 2024-07-09

**Authors:** Panagiotis Avrampos, George-Christopher Vosniakos

**Affiliations:** Manufacturing Technology Laboratory, School of Mechanical Engineering, National Technical University of Athens, Heroon Polytehniou 9, 15773 Athens, Greece

**Keywords:** powder sieving, mesh, vibration, discrete element method, layer quality, roughness, surface coverage, layer thickness, packing density

## Abstract

Powder deposition of even and homogeneous layers is a major aspect of every powder bed fusion process. Powder sieving is commonly performed to powder batches outside of the PBF machine, prior to the part manufacturing stage. In this work, sieving is examined as a method of powder deposition rather than a method to solely filter out agglomerates and oversized particles. Initially, a DEM powder model that has been validated experimentally is implemented, and the sieving process is modelled. The sieving process is optimized in order to maximize mass flow, duration of its linear stage and total mass sieved during linearity. For this, a Taguchi design of experiments with subsequent analysis of variance is deployed, proving that the larger the initial powder loaded in the sieve, the larger the sieve stimulation necessary, both in terms of oscillating frequency and amplitude. The sieve’s aperture shape is also evaluated, proving that the more sides the canonical polygon has, the less the mass flow per aperture for the same maximum passing particle size. Then, the quality of the layer produced via controlled sieving is examined via certain layer quality criteria, such as the surface roughness, layer thickness deviation, surface coverage ratio and packing density. The findings prove that controlled sieving can outperform powder deposition via a non-vibrated doctor blade recoater, both in terms of layer surface quality and duration of layer deposition, as proven by surface skewness and kurtosis evaluation.

## 1. Introduction

Powder sieving is a method used traditionally to filter out agglomerates and control the particle size distribution of a batch of powder. More specifically, by selecting the mesh size of the sieve, the maximum passable particle diameter is controlled. During sieving, the probability of a single particle passing through the mesh during a screening process was defined by Gaudin, and it depends on the effective screening area, the particle diameter to aperture size and the aperture shape [1,2]. Pelevin (2020) expanded the Gaudin probability equation for a particle size range instead of one-size particles and developed a model to calculate the passing possibility, taking into account particle interactions in the sieve [2].

Based on the particle passing probability and the sieving vibrational conditions, the sieving performance is defined. Shimosaka et al. (2000) made an attempt to develop criteria that ensure that the sieve’s excitation is adequate to perform sieving by comparing “the height of particles in the initial packing state to the height of the vibrating particle layer in which 50% of the number of vibrating particles exist” (Shimosaka et al., 2000) [3]. In the literature, DEM simulations have been implemented to examine the effectiveness of both sieving [3,4] and the screening process [5,6], proving the applicability of the method for this study.

Shimosaka et al. (2000), after devising three coefficients (dispersion, existence and velocity coefficients) to ensure that the powder batch is stirred adequately, without increasing powder fugacity to the point where finer particles tend to flee from the sieve, used DEM modelling to obtain an experimentally verified equation that connects sieving rate to vibration amplitude and frequency [3]. However, they only evaluated their model using a dual-size particle batch of glass beads with diameters of 5 mm and 10 mm (mesh size of 9.6 mm). The method remains untested for powder batches characterized by a particle size distribution instead of a uni-size or dual-size powder batch.

Yamane et al. (2012) examined the sieving performance via using DEM simulations for elongated rod-like particles [7]. They examined the vertical and horizontal vibration of a sieve and proved that, for vertical oscillation, sieving rate decreases with increasing particle aspect ratio, while, for horizontal oscillation, sieving rate decreases with aspect ratio. However, no spherical particles were examined in their work [7]. Yoshida et al. (2013) used passage probability models to connect the probability of undersized particles to pass through the sieve’s apertures, by avoiding the oversized ones, to the sieving rate [4].

In PBF processes, this stage of powder treatment has traditionally been performed outside of the PBF machines, by automated sieves that can effectively handle large powder amounts and isolate agglomerates, spatter-produced particles and oversized particles [8]. Then, the sieved powder is fed into the PBF machines, at the feeding bin or silo of the powder deposition mechanism. A feeding silo has been proven to be more beneficial to the commonly used feeding piston, since it can easily accommodate powder condition monitoring systems while providing superior powder economy during the deposition stage [9,10]. A system of powder deposition that utilizes recoater spreading and simultaneous, above the fabrication piston, sieving has been examined in the literature and found to maintain constant particle size distribution along the deposition direction, contrariwise to layers produced via feeding piston-provided powder [9,10]. This was attributed to the constant height of powder accumulation in front of the roller recoater, which maintains a stable pressure and prevents cracks and separations, while the powder segregation is minimized, since fresh powder is fed constantly, and the number of small particles is replenished.

However, despite its undoubted value in terms of particle size distribution control and agglomerate filtering, sieving has never been examined as the sole method of powder deposition. This work suggests the controlled sieving of powder over the desired area in order to ensure that the correct amount of powder is deposited on exactly the right area. It is proven that, with doctor blades or roller spreaders, the smaller particles are compressed and deposited faster, at the start of the layer, while, as the spreader moves towards the end of the layer, the larger particles are dragged along by the spreader [11,12,13], leading to a difference in terms of particle size distribution along the layer itself, an effect known as powder segregation. This is a problem only in the case where a feeding piston is used. If the powder is sprinkled evenly onto the surface from above via a vibrating sieve, and the spreader is then used only to flatten the top of the produced layer, then this problem is countered [9,10]. However, since this is another step adding to the total manufacturing time, it is necessary to optimize sieving and make it as efficient as possible. For this reason, a Taguchi design of experiments (DoE) is used.

In Section 2, the authors describe the materials and methods used in the simulations, define the sieving quality criteria and describe the DoE. Then, the simulation geometry is described, and the Taguchi DoE is explained, together with the variables explained and the quality criteria defined. In Section 3, the results of the sieving simulations are presented. Subsequently, analysis of variance (ANOVA) is used to examine the relations and interactions of the process parameters and their effect on the quality criteria of the sieving process. Furthermore, analysis of these results in Section 4 focuses on the impact of the aperture shape on the sieving performance and the capability of sieving deposition for producing PBF-ready layers. Finally, in Section 5, conclusions are articulated, and suggestions for future work are given.

## 2. Materials and Methods

### 2.1. Powder Modelling and Environment

The powder selected for the experiments was the SA-ZL-20 spherical alumina (Al_2_O_3_) powder provided by the manufacturing company Sinoenergy Group^ΤΜ^ (Beijing, China) [14]. The models and properties used in the simulation can be found in Table 1. It should be noted that powder material properties, particle size distribution and cohesion/adhesion determination methods as well as the physical models that describe the environment are the same as described in previous works [15]. The powder was modelled as spherical particles of a lognormal distribution of mean diameter of 27.488 μm and standard deviation of 23.216 μm, as calculated by the lognormal distribution equation for the D_10_, D_50_ and D_90_ provided by the manufacturer [14]. The maximum and minimum diameters were set at 75 and 5 μm, respectively, to comply with the nominal layer thickness (100 μm) while avoiding extremely fine particles that would drastically increase cohesion.

The alumina bulk material properties and the St.304 material properties can be found in [16] and [17], respectively. For the simulations, a shear modulus value 4 orders of magnitude smaller than its real-world value was selected, which ensured a 67-times smaller simulation time with minimal deviation in terms of results, with the study being on the safe side [15].

The static friction, rolling friction and restitution coefficients were taken from sources in the literature [18], as calculated via the Jenike shear test (ASTM D6773 [19]). The contact model selected was the Hertz–Mindlin with JKR [20,21]. This was the model selected after analyzing the Tabor parameter [22,23] value for the real-world and the shear modulus-adjusted simulation material, after comparison with other potential models, like the Derjaguin–Muller–Toporov (DMT) [24], the Bradley–Derjaguin (BD) [25] and the Maugis–Dugdale (MD) analytical models [26].

The model described incorporates natural gravity and Schiller–Naumann air drag [27] with a motionless air field.

The realistic behavior of the powder model was ensured via cross-check between simulations and experimental measurements of the angle of repose and the angle of avalanche, as can be seen in [18]. The software used for the simulations was EDEM^TM^ (version 2022.2), kindly provided by Altair^TM^ (Troy, MI 48083, USA).

### 2.2. Simulation Bodies and Simulation Description

**Figure 1:**
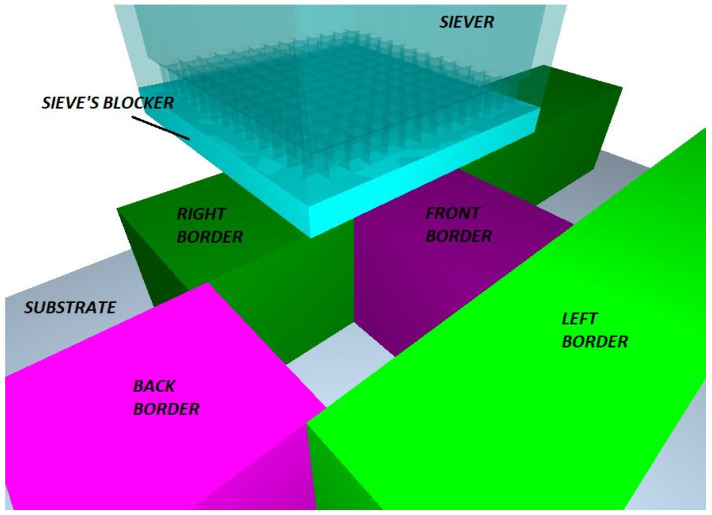
The physical objects of the powder sieving simulation.

The powder sieving simulation features seven (7) bodies, namely the deposition plate, the front, back, left and right border (which define the area in which the sieved powder drops), the sieve (see Table 2 for mesh specs) and the sieve’s blocker (i.e., a plate that blocks the bottom of the sieve). The borders create a square 1 mm/side sample area. The height of the border plates is also equal to 1 mm, while the thickness of the blocker plate is equal to 100 μm (see Figure 1). The model features natural gravity and Schiller–Naumann drag model (see Table 1). All the physical bodies are made of stainless steel 304.

**Figure 2:**
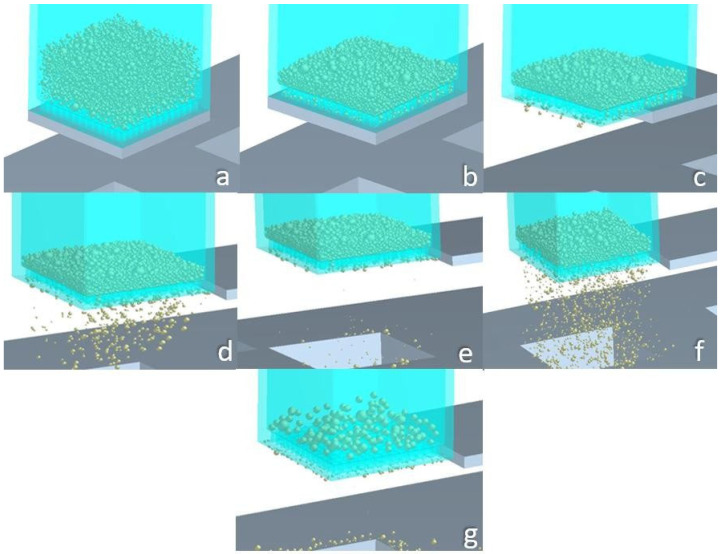
Sieving simulation steps: (**a**) powder generation; (**b**) settled powder; (**c**) blocking plate’s retraction; (**d**) initial “cloud” drops; (**e**) clogging of the sieve’s ducts; (**f**) sieve vibration begins—sieving initiation; (**g**) powder segregation completed.

A virtual body (a 1 mm/side cuboid, enveloped within the sieve) serves as a powder factory. When it is activated, powder instantaneously fills the sieve (see Figure 2a). Its height varies, depending on the trial of the Taguchi DoE, see Section 3.1. Figure 2 depicts the steps of the sieving process.

At the beginning moment of the simulation, all particles are created simultaneously by populating the powder factory cuboid (see Figure 2a) in such a way that inter-particle physical contact is avoided until no other particles can fit into the said virtual body. Then, they free-fall inside the sieve, while the bottom of the sieve remains sealed via the blocking plate. The particles partially occupy the sieve’s ducts, eventually clogging them up (see Figure 2b). When the particles practically become motionless (“frozen” particles), the blocking plate is removed, enabling the particles to fall onto the substrate (see Figure 2c–e). Then, the sieve begins vibrating vertically (Figure 2f) with an amplitude and frequency defined by the Taguchi DoE (see Section 3.1). The simulation is over when particle segregation is complete (see Figure 2g), i.e., when the only particles remaining in the sieve have a size larger than the sieve’s aperture.

The total mass of the particles that have passed through the sieve over time and the particle size distribution of the particles that remain in the sieve over time are extracted. Specific quality criteria for the Taguchi DoE can be defined that use these data. Section 2.3 covers these criteria.

### 2.3. Sieving Phase Identification and Quality Criteria

The sieving process is optimized according to four quality criteria, namely powder mass flow, total powder mass sieved during linearity, sieving duration of linearity and linearity end size ratio (LESR). These criteria will be defined and explained in the following sub-sections.

#### 2.3.1. Powder Mass Flow during Linearity Q (mg/s)

**Figure 3:**
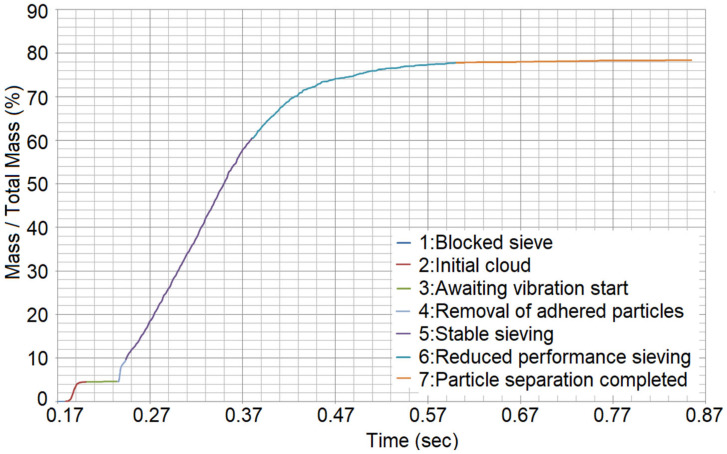
Sieved mass vs. time curve.

It is important to keep the mass flow through the sieve constant, to define the amount of powder that will be deposited onto a specific surface by adjusting the sieving duration. It is desired that the mass sieved and the time are connected via a linear equation. A typical sieved mass versus time curve plot can be seen in Figure 3. The plot refers to Trial #1 from the sieving Taguchi DoE (see Table 3).

Seven (7) sieving stages can be identified, denoted by different colors.

- During Stage 1, the sieve is sealed, hence the powder particles are frozen inside the sieve. However, during the powder generation and the particles’ free fall until this frozen status is achieved, some particles managed to enter the sieve’s ducts before they became clogged down by van der Waals forces acting between the particles.
- When the blocker plate is retracted, the particles that were filling the sieve’s ducts fall onto the substrate, creating an initial “cloud” (Stage 2).
- After these particles fall, no more particles can travel through the ducts while the sieve remains motionless. This is Stage 3, where the sieve is awaiting the vibration in order for the sieving to begin. During this stage, many small particles are hanging from the bottom of the sieve, due to the microscopic adhesive forces that exceed gravity.
- When the vibration begins, at the very first oscillation, these freely hanging particles drop onto the substrate, creating a second “cloud” of particles. This is Stage 4.
- Next, providing that the vibration provides enough energy to enforce the stirring of the powder and facilitate the unclogging of the ducts, the powder begins to pass through the sieve at a steady flow rate (Stage 5); hence, the desired linearity is achieved. During this stage, the fines that remain in the sieve decrease, while the coarse ones tend to remain in the sieve, since they cannot pass through the ducts as easily.
- Eventually, the flow rate decreases, and the linearity of the stable sieve stage is lost (Stage 6). In Stage 6, the segregation continues, even though the sieving rate decreases. The particles that pass through the ducts are infinitesimally smaller than the sieve’s aperture size. This continues until segregation is complete.
- Stage 7 follows, where no more particles pass through the sieve regardless of the sieve’s vibration.

The linearity is desired and ensured in the experiments by a minimum R^2^ value of 0.995 (99.5%), selected by visual inspection of the curve’s shape. It was observed that, at the timestamp when the R^2^ value exceeded 99.5%, Stage 5 (Stable sieving stage) had already ended, and the graph had entered Stage 6 (Reduced performance sieving stage), see Figure 3. This value was selected by trial-and-error to consistently locate the ending of the stable sieving stage. However, whether a large or small mass flow is desired depends on the application. If sieving is used to deposit even, PBF-ready layers without a recoater, it is beneficial that powder sieving is performed slowly, in a controlled manner, with low mass flow rates. Contrariwise, if the deposition is achieved via the simultaneous sieving and spreading of powder via a mechanical recoater (roller or doctor blade), a maximized mass flow is preferrable, to ensure that a sufficient amount of powder to create a slope of constant height in front of the recoater is provided.

**Figure 4:**
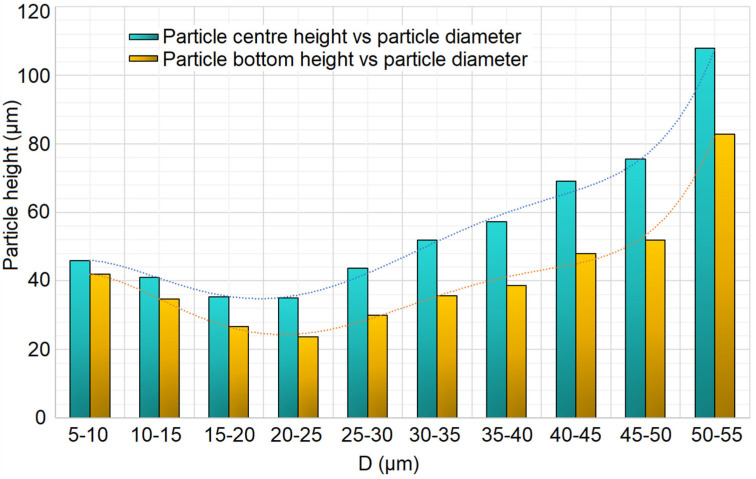
Particle position’s height vs. particle diameter in sieved layer.

Furthermore, when sieving passes beyond the stable phase and into Stage 6 and even Stage 7, the mass flow drastically decreases, while the average size of the deposited particles increases. As a result, at the lowest sections of the layer, the smallest and medium-sized particles are located, while at the highest sections of the layer, the large particles become prevalent, see Figure 4. This shows the average height of the center of the particles in the sieving-produced layer for different particle diameter classes. The larger the particles, the higher their position in the layer, both by particle’s center and particle’s bottom evaluation. The plot refers to Trial #1 from the sieving Taguchi DoE (see Table 3).

This creates problems in all cases, since, if the layer is to be subsequently flattened by a recoating mechanism, the large particles that rest at the top of the layer will be dragged along it, reaching high kinetic energy status due to their direct contact with the recoater, leading to surficial defects. If the layer created by the sieving is to be immediately fused by a laser beam, then the large particles resting at the top will cause surficial defects of the sintered/melted part and can even create large-sized spatter in the case of SLM, since “the larger the particles, the higher likelihood to partially melt and adhere to particles near the part border thus causing a higher surface roughness of the finished part” (Ali et al., 2019) [28]. Hence, maintaining a constant powder mass flow throughout the sieving process will ensure that the particle size distribution will be as even as possible with regard to the layer height, which will lead to high-quality finished parts with homogeneous density and mechanical properties.

#### 2.3.2. Total Powder Mass Sieved during Linearity m (mg)

It must be ensured that a sufficient amount of powder has been sieved onto the surface. Hence, it is necessary to maximize the mass of powder that passes through the sieve during the linear sieving stage. For recoater-facilitated deposition, this amount of powder needs to be larger with regard to the powder amount that will constitute the finished layer, since the scraping of the top of the layer unavoidably expels some material from the surface.

#### 2.3.3. In-Sieve Linearity End Size Ratio LESR (D_90_/A.S.)

It is imperative to identify when the sieve needs to be emptied and refilled, or when it is needed to top it up with raw powder without emptying it. In order to do this, “linearity end size ratio” is introduced. The linearity end size ratio (LESR) indicates the ratio of the D_90_ of the particles that remain in the sieve at the moment when linearity ends, divided by the aperture size of the sieve ducts, see Equation (1). D_90_ is selected since it gives an estimation of the average diameter of the coarse particles, rather than D_10_ and D_50_, which correspond to the average diameter of fines and the average particle size of the sample, respectively. The flow rate depends on the percentage of the coarse particles within the sample, justifying this selection. D_90_ is divided by the aperture size in order to provide a normalized parameter.

(1) $$LESR=\frac{D_{90-(in-sieve,at\;lin.end)}}{A.S.}$$

LESR connects the particle size distribution of the particles in-sieve to the moment when the sieving’s linear behavior ends. Hence, LESR is an indication of the degree of the achieved segregation of the powder sample at the end of the linear sieving stage. It is useful to identify how the sieving parameters interact with this quantity, in order to identify when it is necessary to refill or empty the sieve. Evaluating this leads to powder economy, since, at higher LESR values (close to, or even larger than 1), the vast majority of the particles that remain in-sieve at linearity-end are the ones that physically cannot pass through the apertures of the sieve, so the remaining powder can be disposed of, and the sieve can be refilled with a new powder batch.

Note that refilling the sieve at the linearity-stop moment without emptying the remaining powder might have a negligible effect on powder kinematics if it is done a few times, but it can vastly alter the initial particle size distribution when done multiple times, as the large particles from each refilling batch add up, moving the distribution to the right side. It is strongly suggested that the remaining powder is emptied after every sieving, or, for time efficiency, at least after depositing each layer completely (in multi-material deposition).

#### 2.3.4. Sieving Linearity Duration t_lin_ (s)

As mentioned earlier, depending on the nature of the process, it is important to define the duration of the linear phase of sieving, since, in some cases, a longer linear phase with smaller mass flow is preferable, and in others, a shorter linear phase with larger mass flow is the way to go.

However, this criterion is directly connected to the first two (mass flow Q and total mass m), since, in linearity, Equation (2) applies:

(2) $$Q=\frac{m}{t_{lin}}$$

This means that it is only necessary to examine two out of these three parameters to adequately define the sieving process and its behavior during its linear, stable phase.

## 3. Simulation Experiments

### 3.1. Design of Experiments

**Figure 5:**
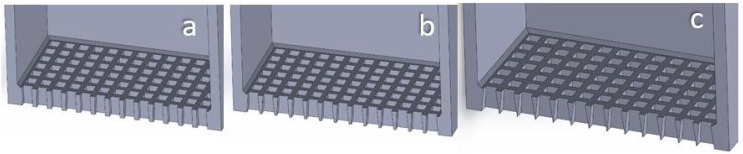
Section cut view of square-aperture sieves with different taper angle: (**a**) 1°; (**b**) 4°; (**c**) 7°.

**Figure 6:**
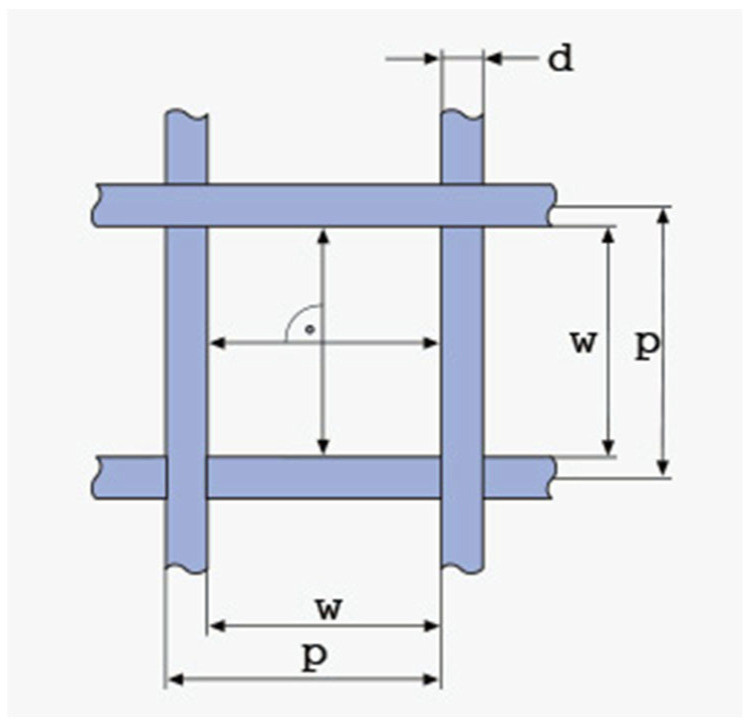
Square-aperture mesh geometry.

The Taguchi DoE was selected to reach conclusions with a robust, statistically sound method while minimizing the number of trials necessary. The quality criteria set beforehand are the ones described in Section 2.3.1, Section 2.3.2, Section 2.3.3 and Section 2.3.4. Four variables are being examined, namely:

- Vibrational frequency of the sieve along the vertical (z) axis, f_vib_ (Hz), examined at three levels: 200 Hz, 350 Hz, 500 Hz. These frequencies were selected in order to be much lower compared to ultrasound sieving, since, at very high frequencies, the continuous offering of very high energy values to the powder leads to an excessive fugacity of the particles, which form a “micro-suspended” state on the sieve [29]. In order to examine this, a very large domain in combination with top-sealed sieves would be necessary to prevent the particles from escaping. The levels selected are mechanically achievable. The minimum level (200 Hz) was selected after preliminary simulations showed that frequencies below 150 Hz were unable to facilitate the necessary stirring of the powder to perform the sieving. In these cases, the sieve would oscillate vertically and carry the powder along with it, leaving the particles motionless with regard to the sieve.
- Vibrational amplitude of the sieve along the vertical (z) axis, A_vib_ (μm), examined at three levels: 20 μm, 35 μm, 50 μm. These values were selected by preliminary trials. It was proven that amplitudes smaller than 10 μm were unable to facilitate the necessary stirring of the powder, while amplitudes larger than 70 μm would cause the powder to escape the 2 mm high sieve, hence leaving the domain of the simulation. These limits must be checked prior to simulations for different powder materials and particle size distributions, since the cohesive forces will be affected, and different excitation levels will be necessary to induce stirring.
- Sieve ducts’ taper angle, θ_tap_ (degrees), examined at three levels: 1°, 4°, 7°. The sieve is open at the top, rectangular, with square apertures. The side of the sieve is 1 mm, and its height is 2 mm (referring to sieve interior dimensions; sieve volume capacity is 2 mm^3^). The thickness of the sieve is 100 μm and kept stable for all the simulations. The ducts have an inverted taper shape; hence the aperture diverges while moving downwards (see Figure 5). This variable aims to examine whether the adhesive forces and duct clogging differ with various taper values. The values chosen were in agreement with industrial manufacturing standards [30], while ensuring that the inverted taper-shaped ducts would not intersect each other throughout the metal sheet’s thickness. The sieving plate is designed by the process of electroforming a thin, 100 μm thick metal sheet to create inverted taper-shaped apertures throughout the sheet. The geometry of the square mesh is described in Figure 6. The figure refers to woven wire mesh, but the same terminology and definitions apply to a sheet form mesh as well [31]. The sieve used in the simulations (see Figure 6) is designed to be a 325 mesh (325 lines/inch) with an aperture width of 50 μm (w = 50 μm) and a mesh pitch of 78.2 μm (p = 78.2 μm). This means that the distance between two consecutive apertures is equal to d = p − w ⇒ d = 28.2 μm. The calculations are in accordance with the specifications provided in [30], as can be seen in Table 2. There is also the possibility of having hexagonal (coxcomb) or circular (round) apertures. Possible differences in their behavior will be examined with regard to the Taguchi-defined optimum levels for the square-aperture sieve, for time reasons, since sieving simulations are quite time-costly.
- Box factory height, h_bf_ (mm), examined at three levels: 0.5 mm, 1.0 mm, 1.5 mm. It determines the amount of the powder that fills the sieve at the beginning of the simulation. As more and more powder stacks into the sieve, the height of the powder stack increases. This means that more particles are stacked onto the ones that are positioned right above the ducts, transferring their weight onto them. Hence, a stronger vibrational excitation will be necessary to facilitate the stirring of the powder that is needed for the sieving to be performed. These levels were selected by trial and error, ensuring that the aforementioned vibrational amplitude and frequency levels would provide adequate stirring of this amount of powder for sieving to be performed. This variable aims to examine how much powder can be fed into the sieve before the stacked weight of the powder particles prevents the sieving from being performed in an acceptably consistent and fast manner. These levels were selected to be in agreement with the selected sieve height (2 mm). These refer to the 25%, 50% and 75% of the height of the sieve.

Table 3 presents the L27 Taguchi orthogonal array for the powder sieving process.

**Table 2:** Mesh specs of the selected square-aperture sieve.

Lines (Line/Inch)	Mesh Pitch (μm)	Maximum Sheet Size (mm)	Aperture Accuracy Guaranteed Area		Min-Max (μm) Aperture Size
**325**	78.2	380	±2	≤Φ180	8–62
			±4	≤Φ300	

**Table 3:** L27 Orthogonal array of the Taguchi DoE for powder sieving and pertinent results.

*TRIAL #*	f_vib_ (Hz)	A_vib_ (μm)	θ_tap_ (deg.)	h_bf_ (mm)	Q (mg/s)	m (mg)	LESR	t_lin_ (s)
***1***	**200**	**20**	**1**	**0.5**	1.141	0.183	0.804	0.171
***2***	**200**	**20**	**4**	**1.0**	0.888	0.238	0.509	0.271
***3***	**200**	**20**	**7**	**1.5**	0.900	0.218	0.398	0.243
***4***	**200**	**35**	**1**	**1.0**	0.844	0.429	0.912	0.523
***5***	**200**	**35**	**4**	**1.5**	0.737	0.659	1.001	0.923
***6***	**200**	**35**	**7**	**0.5**	1.082	0.152	0.661	0.145
***7***	**200**	**50**	**1**	**1.5**	0.576	0.296	0.469	0.501
***8***	**200**	**50**	**4**	**0.5**	0.750	0.110	0.596	0.147
***9***	**200**	**50**	**7**	**1.0**	0.673	0.341	0.787	0.523
***10***	**350**	**20**	**1**	**1.0**	0.771	0.427	0.919	0.578
***11***	**350**	**20**	**4**	**1.5**	0.584	0.664	1.020	1.183
***12***	**350**	**20**	**7**	**0.5**	1.075	0.146	0.642	0.140
***13***	**350**	**35**	**1**	**1.5**	0.404	0.566	0.851	1.388
***14***	**350**	**35**	**4**	**0.5**	0.594	0.102	0.645	0.176
***15***	**350**	**35**	**7**	**1.0**	0.536	0.334	0.813	0.640
***16***	**350**	**50**	**1**	**0.5**	0.372	0.083	0.626	0.233
***17***	**350**	**50**	**4**	**1.0**	0.334	0.269	0.756	0.811
***18***	**350**	**50**	**7**	**1.5**	0.307	0.462	0.765	1.525
***19***	**500**	**20**	**1**	**1.5**	0.408	0.648	1.000	1.622
***20***	**500**	**20**	**4**	**0.5**	0.983	0.111	0.578	0.115
***21***	**500**	**20**	**7**	**1.0**	0.696	0.359	0.811	0.535
***22***	**500**	**35**	**1**	**0.5**	0.496	0.076	0.586	0.159
***23***	**500**	**35**	**4**	**1.0**	0.421	0.282	0.759	0.681
***24***	**500**	**35**	**7**	**1.5**	0.338	0.522	0.830	1.523
***25***	**500**	**50**	**1**	**1.0**	0.278	0.265	0.746	0.969
***26***	**500**	**50**	**4**	**1.5**	0.239	0.452	0.788	2.025
***27***	**500**	**50**	**7**	**0.5**	0.347	0.065	0.582	0.205

### 3.2. Results

**Figure 7:**
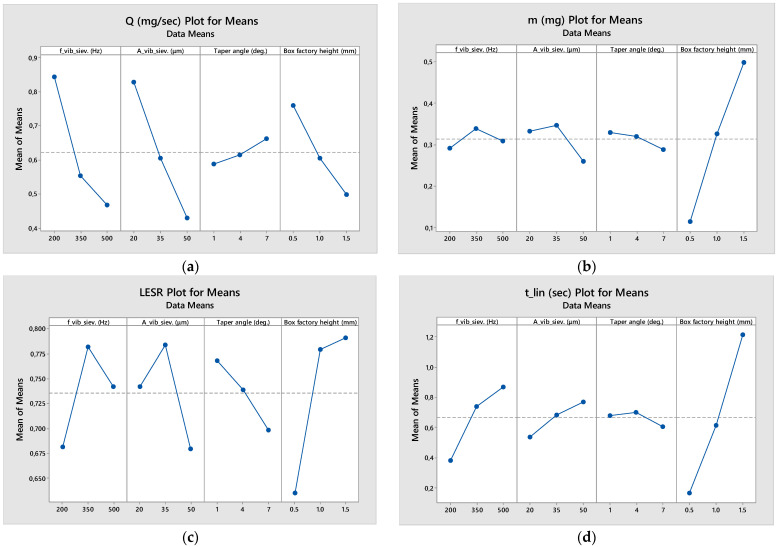
Means plots of the four quality criteria of the powder sieving Taguchi analysis: (**a**) Q (mg/s); (**b**) m (mg); (**c**) LESR; (**d**) t_lin_ (s).

**Figure 8:**
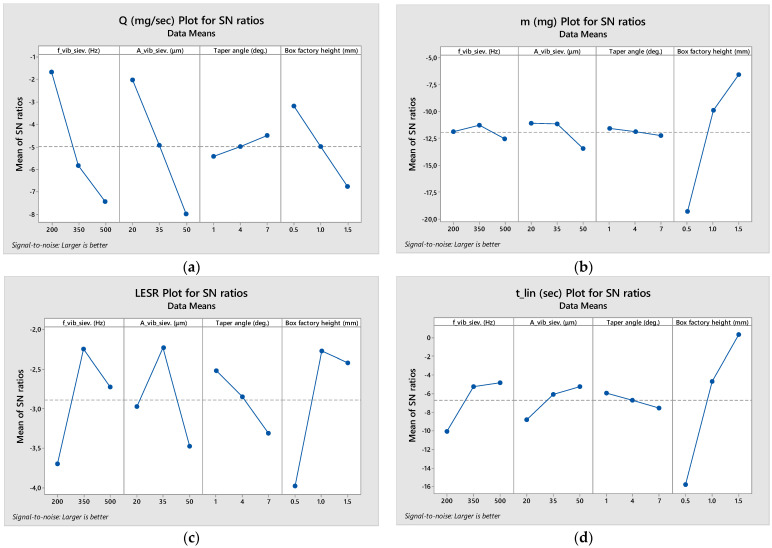
Signal-to-noise ratio of the four quality criteria of the powder sieving Taguchi analysis: (**a**) Q (mg/s); (**b**) m (mg); (**c**) LESR; (**d**) t_lin_ (s).

The results are presented in Table 3 (raw numbers) as well as in Figure 7 (means plot) and Figure 8 (signal-to-noise ratio plot).

Judging from Figure 7, initially it becomes obvious that the total mass of powder that passes through the sieve during the stable phase increases with an increase in the box factory height. The effect of the other three variables is negligible compared to h_bf_. It seems that the optimum is to have a small taper angle and a medium frequency and amplitude as vibration settings. However, in terms of mass flow, the fastest sieving process is performed by implementing low vibrational frequency and amplitude, a large taper angle and the minimum box factory height level. This proves that, the fuller the sieve becomes, the more the sieving process is slowed down, since the stacked-up weight of the powder particles prevents adequate vibrational stirring. The combination of the two aforementioned observations comes in agreement with the plot for the time duration of the linear stage of sieving. Indeed, the larger the vibrational frequency and amplitude and the larger the box factory height, the longer the duration of the linear stage of sieving. Hence, in order to make it a time-efficient process, low frequencies and amplitudes must be selected, and the sieve should be filled with the smallest amount of powder possible, while, if a smooth, long-lasting sieving process is desired, the sieve should be filled with plenty of powder, and high frequency and amplitude levels should be chosen. Finally, LESR attains maximum value for medium frequency and amplitude levels, small taper angle and high box factory height levels.

The higher the LESR value at the end of linearity, the higher powder economy is achieved, since it is strongly suggested that the sieve is emptied and refilled at the end of each sieving pass to achieve uniform particle size distribution in the deposited layer(s) and/or sublayers. A smaller taper angle could mean that the adhesive forces are stronger in the ducts, since the particles are closer to the duct wall throughout their travel from the top to the bottom of the aperture. This would promote faster clogging of the ducts, leading to the ending of the linearity phase and to a slower, non-linear sieving rate.

#### 3.2.1. Mass during Linearity (m)

**Figure 9:**
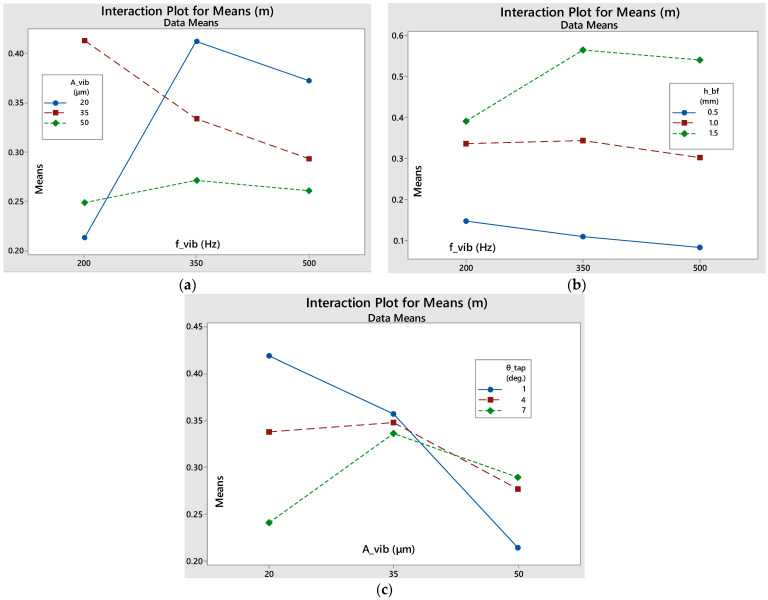
Interaction plots for means of the total sieved mass during the linear sieving stage: (**a**) f_vib_-A_vib_; (**b**) f_vib_-h_bf_; (**c**) A_vib_-θ_tap_.

As seen in Figure 9, the h_bf_-f_vib_ interaction features relatively parallel lines, meaning that there is small interaction between them. However, the f_vib_-A_vib_ interaction graph shows that, at the smallest frequency level, both high and small amplitude levels have similar, relatively bad, results. However, a medium amplitude level corresponds to vastly improved results, indicating that a resonance frequency is being approached. Additionally, for medium and high frequency levels, the lowest amplitude gives the best results. Furthermore, when it comes to the A_vib_-θ_tap_ interaction, even though for low amplitudes, the smaller the taper angle, the better the results, this trend is completely reversed for larger amplitude levels. This potentially derives from the fact that high amplitudes lead to higher reverse bouncing of the particles that are already positioned inside the ducts during the downward motion of the sieve’s oscillation. This leads to more intense clogging in the narrower ducts, while the wider ones can easily be unclogged during the upward motion of the sieve. The box factory height dominates the response of mass sieved in linearity, at 72.09% (see Table A1). Interestingly, interactions contribute more than the other factors.

Equation (3) shows the regression of the total mass sieved in linearity.

(3) $$m=0.173+0.000097f_{vib}-0.00138A_{vib}-0.0483\theta_{tap}+0.197h_{bf}-0.000016f_{vib}A_{vib}+0.000536f_{vib}h_{bf}+0.001182A_{vib}\theta_{tap}$$

The optimum level combination to maximize the mass sieved in linearity is the following: {f_vib_ = 500 Hz, A_vib_ = 20 μm, θ_tap_ = 1°, h_bf_ = 1.5 mm}, which, according to Equation (3), gives the optimum sieved mass amount of m_opt_ = 0.701 mg. After running a cross-checking simulation with the optimum parameter levels, validation of the expected sieved mass amount was achieved, with the value being m_opt, sim_ = 0.648 mg, having a (−) 7.6% deviation from the expected value of 0.701 mg, which is less than the error of the regression equation, as calculated by the ANOVA (15.42%).

Despite these calculations, we notice in Table 3 that there are a few trials, namely #5 and #11, that show a higher mass value (0.659 and 0.664 mg, respectively) than the estimated optimum one. This happens because of the error of the regression. However, since the deviation between these values is less than 2.5%, it is safe to keep the optimum parameter level calculated by the regression.

#### 3.2.2. Mass Flow in Linearity (Q)

Table A2 shows that the vibration frequency and amplitude have a high impact on the flow rate, with a 33.49% and 37.25% contribution, respectively. The box factory height follows, at 16.10%. There is a strong influence of the interaction between amplitude and box factory height, at 4.04%.

Equation (4) shows the regression of the mass flow of the linear sieving stage.

(4) $$Q=1.944-0.000965f_{vib}-0.02101A_{vib}+0.028\theta_{tap}-0.571h_{bf}-0.000008f_{vib}A_{vib}+0.01067A_{vib}h_{bf}-0.0157\theta_{tap}h_{bf}$$

The optimum level combination to maximize the mass flow of the linear stage is the following: {f_vib_ = 200 Hz, A_vib_ = 20 μm, θ_tap_ = 7°, h_bf_ = 0.5 mm}, which, according to Equation (4), gives the optimum mass flow of Q_opt_ = 1.261 mg/s. After running a cross-checking simulation with the optimum parameter levels, validation of the expected mass flow was achieved, with the value being Q_opt, sim_ = 1.181 mg/s, having a (−) 6.3% deviation from the expected value of 1.164 mg/s, which is less than the error of the regression equation, as calculated by the ANOVA (7.14%).

**Figure 10:**
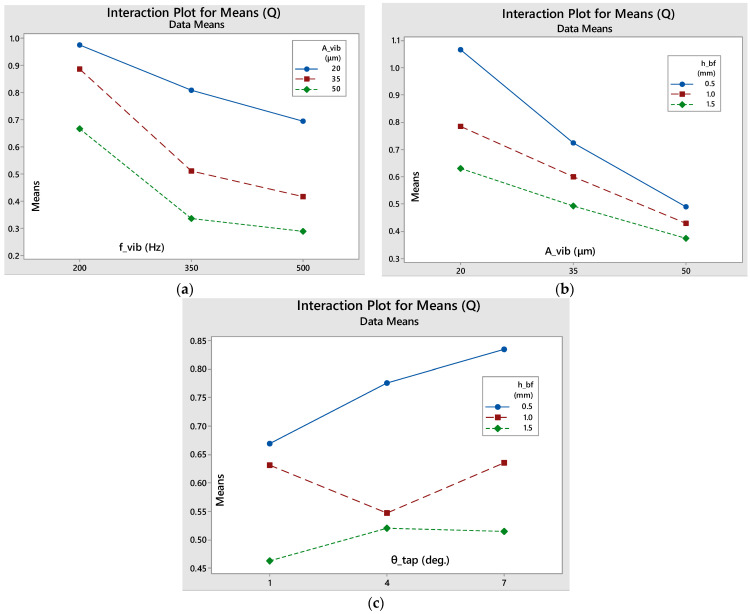
Interaction plots for means of the mass flow during the linear sieving stage: (**a**) f_vib_-A_vib_; (**b**) A_vib_-h_bf_; (**c**) θ_tap_-h_bf_.

The interactions plot for the mass flow, see Figure 10, shows that there is minimal interaction between the parameters, since the lines are relatively parallel in all three graphs.

#### 3.2.3. Duration of Linearity (t_lin_)

Table A3 shows that the box factory height (primarily) and the vibration frequency (secondarily) have a high impact on the duration of sieving linearity, with a 62.75% and 13.55% contribution, respectively. Their interaction also demonstrates a high influence, at 12.84%. The contribution of the other parameters and interactions is insignificant, so it is safe to state that the frequency and the amount of raw powder fed into the sieve are the factors that largely determine the duration of the linear sieving phase.

Equation (5) shows the regression of the time duration of the linear stage.

(5) $$t=0.707-0.001914f_{vib}-0.01061A_{vib}-0.0905\theta_{tap}-0.518h_{bf}+0.003539f_{vib}h_{bf}+0.002235A_{vib}\theta_{tap}+0.00938A_{vib}h_{bf}$$

The level combination that maximizes the duration of linearity is the following: {f_vib_ = 500 Hz, A_vib_ = 50 μm, θ_tap_ = 7°, h_bf_ = 1.5 mm}, which, according to Equation (5), gives the maximum steady sieving duration of t_max_ = 1.948 s. After running a cross-checking simulation with the previous parameter levels, validation of the expected maximum linearity duration was achieved, the value being t_max, sim_ = 1.905 s, having a (−) 2.2% deviation from the expected value of 1.948 s, which is smaller than the error of the regression equation calculated by ANOVA (5.31%). Again, there is a trial, namely #26, that has a larger linearity duration, at 2.025 s. However, this deviation (+4%) is smaller than the regression’s error, so it can be safely assumed that the correct parameter combination has been selected. These deviations show that there are more parameters that possibly affect the sieving time that have not been taken into consideration; however, the deviation from the expected error is reasonably small to make it acceptable.

**Figure 11:**
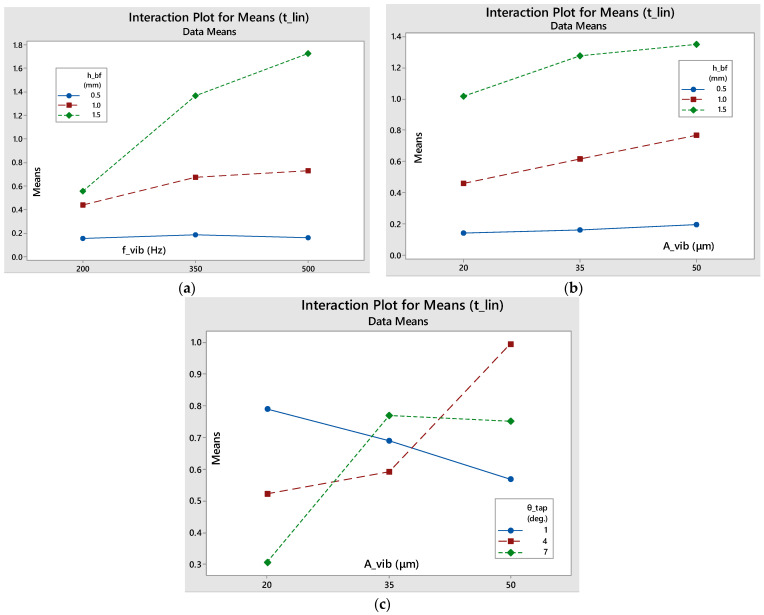
Interaction plots for means of the duration of the linear sieving stage: (**a**) f_vib_-h_bf_; (**b**) A_vib_-h_bf_; (**c**) A_vib_-θ _tap_.

It can be noted that, based on the interaction plots in Figure 11, the amplitude and the amount of powder fed into the sieve do not seem to have any significant interaction, while, on the contrary, amplitude and taper angle interaction seems to be more important, as the lines intersect each other on the intersection plot. Finally, as the vibrating frequency increases and the more powder that is fed into the sieve, the longer the stable phase will last. If the minimum level of powder is fed into the sieve, the steady phase will last approximately the same regardless of the frequency variation, while, at medium and high box factory height levels, the linearity duration increases with the increase in frequency, with the slope increasing as the initial raw powder amount is increased.

In the literature, it can be noted that dry sieving is possible for powder of average diameters as small as 30 μm [32,33]. However, as proven in this work, dry sieving can work for smaller particles with minimal aperture blinding, as long as sufficient vibration is provided to counter the adhesive and cohesive forces and break down the agglomerates. Of course, this only applies up to a limit, since particle diameter in the simulations in this work was at minimum 5 μm, and on average 21 μm, i.e., not much smaller compared to the 30 μm limit that is encountered in the literature, so the results come in agreement with the technological limitations of the method. It should be taken under consideration that the vibration frequencies selected were larger compared to the common ones used (200, 350 and 500 Hz instead of the commonly used 10–50 Hz of industrial sifting machines [7]) in order to avoid aperture clogging without drastically increasing the powder fugacity, which happens at ultrasonic frequencies (approx. 36,000 Hz) [29].

## 4. Analysis and Discussion

### 4.1. Aperture Shape Influence

In order to evaluate the impact of the aperture shape (square, hexagonal or circular) on the aforementioned sieving performance criteria, it was decided that, for time economy, the shape effect only would be examined on the three optimum sieving parameter sets, i.e., the one that maximizes mass flow, the one that maximizes sieved mass and the one that maximizes duration of the linear sieving stage.

**Figure 12:**
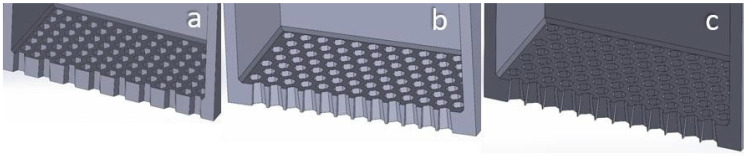
Section cut view of the hexagonal-aperture sieves. Sieve thickness is 100 μm. Inverted taper angle: (**a**) 1°; (**b**) 4°; (**c**) 7°.

**Figure 13:**
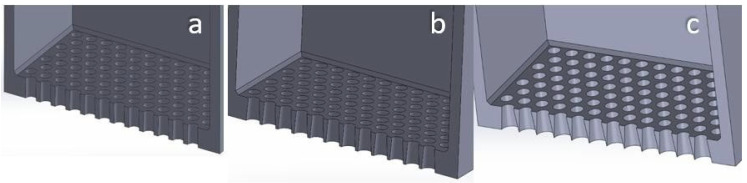
Section cut view of the circular-aperture sieves. Sieve thickness is 100 μm. Inverted taper angle: (**a**) 1°; (**b**) 4°; (**c**) 7°.

The ideal sieving sets of parameter levels are the following, see Table 4:

In order to examine solely the shape effect, the designed (regular) hexagonal aperture (see Figure 12) has a distance between facing edges equal to the side of the square aperture (50 μm). This leads to a side equal to 28.868 μm. Similarly, the circular aperture’s diameter (see Figure 13) is equal to the side of the square aperture. In this way, it is ensured that all the designed sieves allow particles of the same maximum size to pass through their ducts. An alternative design would be to opt for apertures of different shape, but define their characteristic dimensions by ensuring that the apertures maintain a constant surface area. However, in powder deposition for PBF-ready layers, it is of utmost importance to control the maximum particle diameter, ensuring that it is smaller than the theoretical layer thickness. Hence, the author opted for the aperture design method that provides accurate particle size “filtering”.

Additionally, the distance between neighboring apertures is kept the same regardless of the aperture’s shape. A constant inter-aperture distance was selected in order to maintain the same amount of interference between neighboring apertures among all the different sieve designs. Decreasing the distance would increase the interference by neighboring apertures and vice versa. This way, the calculated aperture-to-total area ratio is 42.25% for the square aperture sieve, 40.8% for the hexagonal-aperture sieve and 36.9% for the circular-aperture sieve, given that the total surface area of the sieve is equal to 1 mm^2^. The details of this calculation can be seen in Table 5.

Figure 5, Figure 12 and Figure 13 show the geometry of the sieves used in the simulations.

If a spherical particle of 50 μm diameter would try to pass through the defined apertures, in the case of the square aperture, it would have 4 contact points to it; in the case of the hexagon, the number of contact points would be 6; while in the case of the circular aperture, the particle would make full contact with the top edge of the aperture. The more the number of contact points, the more intense the friction phenomena when the particle attempts to pass through the aperture. Hence, the circular apertures would not allow the larger particles to pass. A particle size distribution of the powder in the deposited layer slightly moved towards the left would be expected as we move from square towards hexagonal and circular aperture sieves. For the same reason, the LESR of the powder inside the sieve at the end of linearity would slightly decrease, as some smaller particles with diameters slightly smaller than 50 μm will find it tougher to pass through the hexagonal and circular apertures. The mass sieved and the mass flow are expected to decrease as well.

In Table 6, the main quality indicators for the sieving process are compared for different aperture shapes in the three trials with parameter combinations that maximize sieved mass, mass flow and linearity duration. These were calculated after running sieving DEM simulations using the variable level sets of Table 6. The percentages are calculated with regard to the respective value of the same parameter set for the square aperture. The following observations can be made.

In the case of maximizing the powder mass that has passed through the sieve in linearity, the mass itself remains relatively unchanged, at approximately 0.636–0.661 mg. The same applies for LESR, at 1.303–1.334. However, the linearity duration decreases by 13.4% in the square-to-hexagonal transition, and then returns to a value similar to the one of the square aperture for the circular-aperture trial. Of course, since the mass is relatively stable, the opposite with regard to the linearity duration applies for the mass flow. This contradicts the expected result that was stated above. The experiment indicates that there is no strong statistical difference between the square-aperture and circular-aperture sieve performance (see Table 6), as can be judged by the percentages provided, but the hexagonal-sieve performance shows higher mass flow and decreased linearity duration.

In the case of maximizing the powder flow of the linear phase of sieving, no statistically important differences can be observed in any of the set quality indicators, namely mass flow, mass, linearity duration and LESR. Any variances among these can be attributed to slight differences during the powder generation phase, as the particles are generated randomly by a lognormal distribution, as explained in the powder spreading-relevant chapter of this work. The powder generation happens again at the start of each trial, and differences of approximately 2.9% in total powder mass have been observed between the three trials of the mass flow-maximizing set.

In the case of maximizing the duration of linearity, when moving from a square-aperture sieve to a hexagonal-aperture sieve, the mass flow decreases by 8.4%. However, the total mass sieved remains almost the same, as well as the LESR. This means that the decreased mass flow is the only factor affecting the duration of linearity, which is increased by 10.9%. However, the total aperture area of the hexagonal-aperture sieve is only 3.4% smaller with regard to the one of the square-aperture sieve. This means that the decrease in the mass flow would be expected up to a 3.4%, and the remaining 5% can be attributed to the decreased passing probability for the larger particles to pass through the sieve ducts. When we move from the hexagonal to the circular aperture sieve, the mass flow further decreases by another 6.2% with regard to the square one. However, in this case, the linearity duration also decreases with regard to the square one by 3.1%. This is attributed to the fact that, when linearity stops, the LESR value is 6.4% lower compared to the square sieve. This makes the total mass sieved also decrease by 16.7%, while the hexagonal sieve allowed approximately the same mass as the square one. Hence, the more the aperture shape deviates from the square shape and moves towards regular polygons of an increasing number of sides, at first the mass sieved is not affected, and the only values affected are the mass flow and linearity duration, but after reaching a critical number of sides, this trend ceases to exist, and the LESR value decreases, causing even the duration of linearity to decrease.

The differences in the observations between the three trials clearly show that the aperture shape is an important factor that demonstrates interaction with the four variables of the experiment.

The last column of Table 6 provides an important insight. If the mass flow becomes normalized by the number of apertures for each sieving trial (Q_n_ = Q/N_a_), then it becomes clear that, regardless of the sieving variable levels, as we move from the square towards the circular apertures, the mass flow per aperture decreases. This comes in complete agreement with the prediction, since

- It is increasingly difficult for borderline passing particles (i.e., particles of 50 μm diameter) to penetrate the apertures and move through them, and
- Square apertures, while a borderline passing particle passes through them, leave some space at the corners for fine particles to pass. This space becomes smaller for hexagonal apertures and becomes non-existent for circular apertures.

Hence, for sieves that possess apertures that correspond to borderline passing particles of the same size, it is important to increase the number of apertures while moving from the square aperture shape towards the circular aperture shape in order to achieve the same mass flow.

### 4.2. Sieved Layer Quality

As mentioned earlier, in the case of sieving as a preparatory step to powder deposition via a recoating mechanism, such as a doctor blade or a counter-rotating roller, it is imperative that the powder amount necessary is sieved as fast as possible to minimize the total manufacturing time. In this case, sieving only functions as a method to prevent agglomerates and large powder particles from being deposited in the layer. However, it is possible that sieving can function as a powder spreading mechanism. In that case, it is imperative that the powder deposition be slow and controllable, since it needs to stop exactly at the right moment, i.e., when the thickness of the layer is the desired one. In this work, we will examine the deposited layer quality for layers produced solely by sieving, and they will be compared in terms of quality to the optimized layers with and without blade vibration that were calculated in the previous chapter.

It is very important to examine whether controlled sieving is able to produce PBF-appropriate layers of powder, since this would be a very time-efficient powder deposition method that would promote powder economy. In order to do this, the same Taguchi DoE as the one used in Section 3.1 (Table 3) is used; however, the quality criteria will be the ones used in [15], i.e., layer thickness deviation (LTD), surface coverage ratio (SCR), true packing density (PD_tr_) and root-mean-square roughness (S_q_-RMS). The deposited layer will be considered ready for evaluation at the precise moment when the mass inside the sample square will be equal to the optimum spread layer mass via a doctor blade (SCR-RMS), i.e., equal to 0.205 mg [15]. By comparing the true packing density of the spread layers created via a vibrated doctor blade recoater for a Taguchi DoE in [15] to the true packing density values of Table 7, it becomes obvious that it remains stable regardless of the deposition method (with doctor blade recoater, or recoater-less, via sieving). Hence, the actual layer thickness is expected to be similar by maintaining a stable total layer mass, at 0.205 mg [15]. The design and results of the Taguchi DoE for the powder deposition via sieving can be seen in Table 7. The levels of the sieving variables were kept the same as they were set in Section 3.1 (Table 3), in order to make consistent observations that connect sieving performance criteria to deposited layer quality.

After proving that skewness (Ssk) and kurtosis (Sku) are general layer surface quality indicators that take under consideration LTD, SCR and RMS roughness [15], these two quantities will be used to evaluate the quality of the powder layers created via sieving.

The layers developed have negative skewness, since the spherical powder particles create distributions that deviate to the upper side relative to the mean line. The developed surfaces are also typically leptokurtic, i.e., with a kurtosis value larger than 3, since sharpness of the profile is developed by the valleys, which are steeper and narrower the higher the layer quality is. The steeper and narrower the valleys, the higher the Sku value. Furthermore, the flatter and larger the “plateaus” of the surface, the smaller the (negative) Ssk value. Thus, the more packed the particles are on the top of the layer, the flatter the plateaus will appear to be, and the smaller the (negative) surface skewness value [34].

The layers developed have negative skewness, since the spherical powder particles create distributions that are deviated to the upper side with regard to the mean line. The developed surfaces are also typically leptokurtic, i.e., with a kurtosis value larger than 3, since the only sharpness of the profile is developed by the valleys, which are steeper and narrower the higher the layer quality is. The steeper and narrower the valleys, the higher the Sku value. Furthermore, the flatter and larger the “plateaus” of the surface, the smaller the (negative) Ssk value. This means that the more packed the particles are on the top of the layer, the flatter the plateaus will appear to be, and the smaller the (negative) surface skewness value.

In Table 8, a comparison between the powder deposition via doctor blade-facilitated spreading with the powder deposition via controlled sieving is presented. Note that ‘reg’ stands for “regression”, while ‘sim’ for “simulation”. The zero-vibration trial cannot have regression values calculated, since this level was not included in the Taguchi design. From this, it can be deduced that despite the fact that sieving-facilitated powder deposition leads to a layer quality inferior to the one achieved via a vibrated doctor blade recoater, the layer quality, however, is superior to the one achieved by a non-vibrated doctor blade recoater.

**Figure 14:**
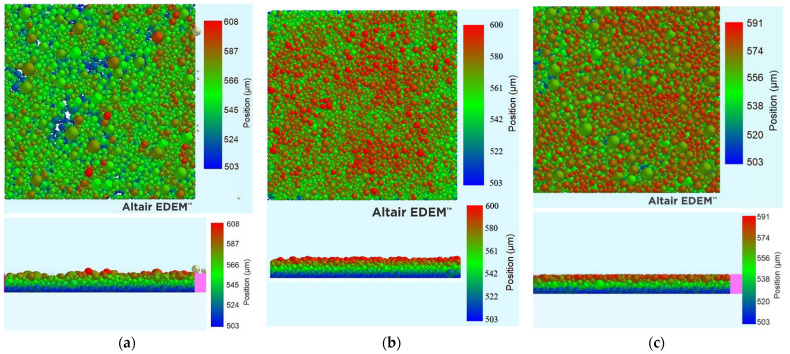
Visual comparison of the optimum layers developed by (**a**) vibration-less doctor blade; (**b**) controlled sieving; (**c**) vibrated doctor blade.

A visualization of this comparison is provided in Figure 14. This is a particularly important observation, since most industrial powder deposition systems designed for PBF do not use a vibrating doctor blade, but a simple doctor blade moving only linearly over the substrate, parallel to the fabrication piston. In this case, it would be better to simply use a vibrating sieve, since by controlling properly the vibration frequency, amplitude and powder level inside it, a better surface quality would be achieved in a much lower deposition time, since the time needed would be approximately $t=m_{opt}/Q_{\#26}=0.205\;mg/0.239\;mg/s=0.858\;s<t_{lin,\#26}=2.025\;s$. The data used can be seen in Table 9.

In general, the quality of the deposited layers via sieving largely depends on depositing the necessary powder mass during linearity, i.e., before the small particles become largely depleted and the mass flow becomes a lot smaller. In general, all trials that have a powder factory height of 0.5 mm have powder mass deposited in linearity smaller than the necessary 0.205 mg (see Table 3 and Table 10). This means that in order to deposit the necessary amount of powder, we would need to keep sieving during the non-linear sieving stage. This would lead to sprinkling the top of the layer with particles of larger diameter, leading to higher peaks. This would potentially decrease the LTD, since many particles would exceed the desired height that is defined by the theoretical layer thickness, but the surface roughness would be increased. Furthermore, the kurtosis value would be decreased, since the sharpness of the surface would be decreased. The valleys would grow wider and the plateaus not that extended, alleviating the leptokurtic surface element. The skewness would be increased (in negative values), since the surface would not be that deviated to the top side anymore.

Judging from Table 10, it becomes obvious how, to obtain the best sieved powder layer quality, it is imperative to ensure that the duration of the linear sieving stage in combination with the linearity mass flow are capable of providing the necessary powder mass before the ending of the linear stage. In order to ensure that a good-quality layer is deposited via sieving, the user must select sieving parameters that ensure a sieved mass during linearity greater than the mass of the ideal spread layer via a vibrating doctor blade.

In the previous chapters, the author assumed that slower sieving can be beneficial in order to develop good-quality, PBF-ready powder layers without the need for levelling them via a recoating mechanism. This assumption has been confirmed, since sieving parameter combinations that promote longer linearity duration are in general connected to better deposited layer quality, as seen in Table 10.

The last two columns of Table 10 compare the quality of the examined layer to the one achieved by powder spreading with a non-vibrating doctor blade (see Table 8). In seven out of the nine trials using a powder factory height of 0.5 mm, the results by both Ssk and Sku evaluation were lower than that achieved by a non-vibrating doctor blade recoater. In conclusion, in order to produce layers comparable to the ones via non-vibrating doctor blades, it is necessary to feed the sieve with enough powder, but ensure it is oscillated at a proper level to facilitate the motion of the particles and prevent clogging. Furthermore, time adjustment of the vibration and blocking or unblocking the bottom part of the sieve via a plate at the correct timing are of paramount importance to achieve the desired layer height.

In order to ensure that the minimum necessary powder amount is provided, the total mass of the particles that can physically pass through the sieve ducts can be calculated via the particle size distribution of the powder and the total powder mass fed into the sieve.

In order to estimate the layer quality, the average rating of their Ssk and their Sku value is calculated, the highest rating (100%) being assigned to the best layer and the lowest (0%) to the worst layer. The results, along with the values of every quality criterion described, are shown in Table 11. The results are sorted in increasing order of average rating of the ones that were calculated for Ssk and Sku.

Based on Table 11, the following conclusions are drawn:

- Trials with larger (t_lin_-t_0.205_) and (m-m_des_) values are superior in terms of quality. Hence, trials with h_bf_ = 0.5 mm are the least promising, unless they feature low sieve excitation levels (f_vib_ = 200 Hz and A_vib_ = 20 or 35 μm), see trials #6 and #1. Inversely, trials with h_bf_ = 1.5 or 1 mm and low sieve excitation levels (f_vib_ = 200 Hz and A_vib_ = 20 or 35 μm) create layers of average quality compared to the ones of the same initial powder amount (h_bf_ = 1.5 or 1 mm) combined with high sieve excitation levels (f_vib_ = 500 or 350 Hz and A_vib_ = 50 or 35 μm).
- In continuation from the previous point, the optimum layers are the ones developed by the sieving combination that promotes the highest mass sieved in linearity combined with the lowest mass flow, meaning indeed that the slower the sieving rate, the better the sieved deposited layer quality.
- SCR remains practically unchanged for every trial (99.3 ± 0.1% for the sample of the 27 trials), indicating that the coverage ratio is an intrinsic property of the sieving method in terms of sieving deposition.
- True packing density remains practically unchanged for every trial (67.6 ± 0.5% for the sample of the 27 trials), as was expected. However, slight changes indicate the opposite trend to the expected one, i.e., higher true packing density for the lower quality layers. This can be again explained by the presence of oversized particles on top of the layer. The oversized particles occupy a large space, leading to a slight increase in the true packing density, as +it was defined in [15].
- In certain trials (#12 and #20), the LTD value appears to point towards a higher layer quality, even though the other criteria point towards the opposite. This is, as explained previously, due to the fact that the average height is heavily influenced by extreme values. In both these trials, in order to sieve the desired powder mass (0.205 mg), sieving had to continue past the end of the linear stage. This caused the accumulation of oversized particles on the top of the layer, creating high peaks that decrease the |LTD| value, but simultaneously degrade the layer quality, as depicted by S_q_.
- Sku and Ssk again prove themselves to be accurate layer quality indicators, capable of identifying whether an increase in LTD (decrease in |LTD|), like in trials #12 and #20, points towards an actual or false layer quality increase.

## 5. Conclusions

In this work, Taguchi DoE for the powder sieving process was used to identify the sieving process parameter combinations (vibrational frequency and amplitude, sieve’s taper angle and powder level at the beginning of sieving) in order to maximize the powder sieved, the duration of the sieving’s linear stage and the powder mass flow through the sieve.

- It has been proven that it is not possible to simultaneously maximize the aforementioned criteria.
- The powder fed into the sieve prior to sieving has been proven to be the most impactful among the criteria, since the larger it is, the larger the powder amount sieved during linearity and the linearity duration.
- Smaller frequencies and amplitudes combined with low initial powder level promote higher mass flow, while larger frequencies and amplitudes combined with higher initial powder level promote longer duration of the linear stage.
- The taper angle of the sieve’s apertures is proven to have negligible effects on the selected quality criteria.
- The shape of the sieve’s apertures has significant interactions with the other sieving parameters, which made it impossible to identify certain relations. The authors will look to evaluate this in their future work. However, it became clear that the powder mass flow per aperture decreases regardless of the sieving conditions with the increasing number of sides the canonical polygon-shaped aperture has, given that the maximum passing particle diameter is kept stable.
- Finally, the quality of the powder layer produced via controlled sieving is optimized by ensuring that the duration of the linear sieving stage in combination with the linearity mass flow are capable of providing the necessary powder mass before the ending of the linear stage. Opting for settings that provide high duration of linearity in combination with large powder mass sieved during linearity is strongly recommended.
- The quality of powder layers deposited via controlled sieving is proven to be superior with regard to the quality of the ones deposited by non-vibrated doctor blade recoaters, but worse compared to vibration-assisted doctor blade deposition.

In the future, the authors aim to develop a sieving apparatus that will be able to perform controlled sieving in order to experimentally prove the findings of this work. Once the sieving process is adequately controlled, a novel, recoater-less powder deposition system based on controlled sieving will be developed, with the aim of being installed on industrial SLS/SLM machines to accelerate the powder spreading process.

## Figures and Tables

**Table 1:** Simulation physical models and powder settings.

Powder properties (a-Al_2_O_3_)	Particle size		D_10_ (μm)	8.2
			D_50_ (μm)	21
			D_90_ (μm)	47.5
	Lognormal parameters	Mean	μ_X_ (μm)	27.488
		Std deviation	σ_X_ (μm)	23.216
	Particle size limits	Max diameter	D_max_ (μm)	75
		Min diameter	D_min_ (μm)	5
	Powdermaterial	Density	ρ (kg/m^3^)	3820
		Simulation shear modulus	G_sim_ (Pa)	1 × 10^7^
		Real-world shear modulus	G_rw_ (Pa)	1.29 × 10^11^
		Poisson ratio	ν	0.3
Body material properties (St.304)		Density	ρ (kg/m^3^)	8000
		Shear modulus	G (Pa)	6.23 × 10^10^
		Poisson ratio	ν	0.275
Material interaction coefficients		Al_2_O_3_-Al_2_O_3_ Restitution coef.	C_r, Al-Al_	0.5
		Al_2_O_3_-Al_2_O_3_ Static friction coef.	C_sf, Al-Al_	0.34
		Al_2_O_3_-Al_2_O_3_ Rolling friction coef.	C_rf, Al-Al_	0.05
		Al_2_O_3_-St.304 Restitution coef.	C_r, Al-304_	0.52
		Al_2_O_3_-St.304 Static friction coef.	C_sf, Al-304_	0.2
		Al_2_O_3_-St.304 Rolling friction coef.	C_rf, Al-304_	0.05
Physical models	Contact (Hertz–Mindlin + JKR)	Particle relative approach at tear-off	δ_to_ (μm)	1.451
	Gravity	Acceleration of gravity	$\vec{g}$ (m/s^2^)	$-9.81\hat{k}$
	Air Drag (Schiller–Naumann)	Air density	ρ_air_ (kg/m^3^)	1.225
		Air kinematic viscosity	ν_air_ (Pa∙s)	1.81 × 10^−5^
		Air velocity	$\vec{u_{air}}$ (m/s)	$0\hat{i}+0\hat{j}+0\hat{k}$
		Scale of drag effect (full-scale)	Scale	1

**Table 4:** Ideal sieving parameter sets based on different quality criteria.

Maximum m	Maximum Q	Maximum t_lin_
$\left(\Sigma_{1}\right):\left\{\begin{array}{c} f_{vib}=500\;Hz\\ A_{vib}=20\;\mu m\\ \begin{array}{c} \theta_{tap}=1{}^{\circ}\\ h_{bf}=1.5\;mm \end{array} \end{array}\right\}$	$\left(\Sigma_{2}\right):\left\{\begin{array}{c} f_{vib}=200\;Hz\\ A_{vib}=20\;\mu m\\ \begin{array}{c} \theta_{tap}=7{}^{\circ}\\ h_{bf}=0.5\;mm \end{array} \end{array}\right\}$	$\left(\Sigma_{3}\right):\left\{\begin{array}{c} f_{vib}=500\;Hz\\ A_{vib}=50\;\mu m\\ \begin{array}{c} \theta_{tap}=7{}^{\circ}\\ h_{bf}=1.5\;mm \end{array} \end{array}\right\}$

**Table 5:** Active sieving area for sieves with different aperture shapes. Total aperture percentage is calculated with regard to the square sieve.

	N_a_ (# of Apertures)	Characteristic Dimension	Aperture Area Type	Aperture Area (mm^2^)	Total Aperture Area (mm^2^)
**Square**	169	Side: $a_{s}=50\;\mu m$	$A_{s}={a_{s}}^{2}$	2.5 × 10^−3^	0.4225 (0%)
**Hexagonal**	188	Side: $a_{h}=28.868\;\mu m$	$A_{h}=\frac{3\sqrt{3}}{2}{a_{h}}^{2}$	2.17 × 10^−3^	0.4080 (−3.4%)
**Circular**	188	Diameter: $D_{c}=50\;\mu m$	$A_{c}=\pi {\left(\frac{D_{c}}{2}\right)}^{2}$	1.963 × 10^−3^	0.3690 (−12.7%)

**Table 6:** Aperture shape effect comparison.

Trial	Trial Settings	Aperture Type	Q (mg/s)	m (mg)	LESR	t_lin_ (s)	Q_n_ (mg/s)
**Σ_1_** **(max m)**	$\left(\Sigma_{1}\right):\left\{\begin{array}{c} f_{vib}=500\;Hz\\ A_{vib}=20\;\mu m\\ \begin{array}{c} \theta_{tap}=1{}^{\circ}\\ h_{bf}=1.5\;mm \end{array} \end{array}\right\}$	**Square**	0.408	0.648	1.304	1.622	0.002414
		**Hexagonal**	0.468 (+14.7%)	0.636 (−1.9%)	1.334 (+2.3%)	1.405 (−13.4%)	0.002489
		**Circular**	0.435 (+6.6%)	0.661 (+2.0%)	1.303 (−0.8%)	1.595 (−1.7%)	0.002314
**Σ_2_** **(max Q)**	$\left(\Sigma_{2}\right):\left\{\begin{array}{c} f_{vib}=200\;Hz\\ A_{vib}=20\;\mu m\\ \begin{array}{c} \theta_{tap}=7{}^{\circ}\\ h_{bf}=0.5\;mm \end{array} \end{array}\right\}$	**Square**	1.121	0.193	1.177	0.185	0.006633
		**Hexagonal**	1.150 (+2.6%)	0.180 (−6.7%)	1.192 (+1.3%)	0.165 (−10.8%)	0.006117
		**Circular**	1.089 (−2.9%)	0.194 (−0.5%)	1.194 (+1.4%)	0.185 (≡)	0.005793
**Σ_3_** **(max t_lin_)**	$\left(\Sigma_{3}\right):\left\{\begin{array}{c} f_{vib}=500\;Hz\\ A_{vib}=50\;\mu m\\ \begin{array}{c} \theta_{tap}=1{}^{\circ}\\ h_{bf}=1.5\;mm \end{array} \end{array}\right\}$	**Square**	0.226	0.426	1.045	1.905	0.001337
		**Hexagonal**	0.207 (−8.4%)	0.431 (+1.2%)	1.020 (−2.4%)	2.113 (+10.9%)	0.001101
		**Circular**	0.193 (−14.6%)	0.355 (−16.7%)	0.978 (−6.4%)	1.845 (−3.1%)	0.001027

**Table 7:** L27 Orthogonal array of the Taguchi DoE and results for powder deposition via sieving.

*Trial* *#*	F_vib_ (Hz)	A_vib_ (μm)	θ_tap_ (deg.)	h_bf_ (mm)	LTD (μm)	$\left|LTD\right|$ (μm)	SCR (%)	$S_{q}$ (μm)	${PD}_{tr}$ (%)	${PD}_{th}$ (%)	$S_{a}$ (μm)	Ssk	Sku
***1***	**200**	**20**	**1**	**0.5**	−21.0	21.0	99.3	19.0	67.8	53.7	14.8	−0.788	4.269
***2***	**200**	**20**	**4**	**1.0**	−20.2	20.2	99.3	18.2	67.5	54.0	14.0	−0.786	4.583
***3***	**200**	**20**	**7**	**1.5**	−20.5	20.5	99.3	18.1	67.4	53.7	14.1	−0.794	4.571
***4***	**200**	**35**	**1**	**1.0**	−20.4	20.4	99.3	18.5	67.3	53.7	14.5	−0.745	4.312
***5***	**200**	**35**	**4**	**1.5**	−20.7	20.7	99.3	18.7	67.4	53.5	14.5	−0.747	4.338
***6***	**200**	**35**	**7**	**0.5**	−21.3	21.3	99.3	18.5	68.1	53.7	14.5	−0.732	4.293
***7***	**200**	**50**	**1**	**1.5**	−20.6	20.6	99.4	18.1	67.5	53.7	14.1	−0.682	4.372
***8***	**200**	**50**	**4**	**0.5**	−21.4	21.4	99.3	19.2	68.1	53.7	15.1	−0.650	3.948
***9***	**200**	**50**	**7**	**1.0**	−20.3	20.3	99.4	17.4	67.4	53.8	13.4	−0.800	4.719
***10***	**350**	**20**	**1**	**1.0**	−20.4	20.4	99.4	18.4	67.6	53.9	14.2	−0.834	4.476
***11***	**350**	**20**	**4**	**1.5**	−19.9	19.9	99.4	18.1	67.4	54.0	14.0	−0.770	4.499
***12***	**350**	**20**	**7**	**0.5**	−19.8	19.8	99.4	19.9	67.9	54.6	15.5	−0.400	3.884
***13***	**350**	**35**	**1**	**1.5**	−20.2	20.2	99.3	17.9	67.4	53.9	13.8	−0.832	4.666
***14***	**350**	**35**	**4**	**0.5**	−20.5	20.5	99.3	20.5	68.3	54.4	16.1	−0.455	3.701
***15***	**350**	**35**	**7**	**1.0**	−20.6	20.6	99.4	17.0	67.5	53.7	13.1	−0.809	4.924
***16***	**350**	**50**	**1**	**0.5**	−22.0	22.0	99.3	21.4	68.4	53.4	17.0	−0.482	3.385
***17***	**350**	**50**	**4**	**1.0**	−19.9	19.9	99.4	16.4	67.0	53.8	12.6	−0.864	5.165
***18***	**350**	**50**	**7**	**1.5**	−20.5	20.5	99.3	18.0	67.4	53.7	13.9	−0.873	4.643
***19***	**500**	**20**	**1**	**1.5**	−20.7	20.7	99.3	19.0	67.8	53.8	14.8	−0.784	4.286
***20***	**500**	**20**	**4**	**0.5**	−19.8	19.8	99.3	20.1	67.9	54.6	15.7	−0.421	3.896
***21***	**500**	**20**	**7**	**1.0**	−20.3	20.3	99.3	18.1	67.6	54.0	14.1	−0.802	4.517
***22***	**500**	**35**	**1**	**0.5**	−21.8	21.8	99.3	21.0	68.5	53.6	16.5	−0.536	3.559
***23***	**500**	**35**	**4**	**1.0**	−19.9	19.9	99.4	17.0	66.9	53.7	13.1	−0.851	4.965
***24***	**500**	**35**	**7**	**1.5**	−20.5	20.5	99.4	17.4	67.4	53.7	13.5	−0.865	4.792
***25***	**500**	**50**	**1**	**1.0**	−20.0	20.0	99.5	16.2	67.0	53.7	12.5	−0.839	5.074
***26***	**500**	**50**	**4**	**1.5**	−20.0	20.0	99.4	16.2	67.1	53.8	12.4	−0.951	5.550
***27***	**500**	**50**	**7**	**0.5**	−29.6	29.6	99.2	20.3	68.7	48.5	16.1	−0.430	3.316

**Table 8:** Recoater spreading vs. sieving-produced layer comparison.

Method	$u_{tr}$ (m/s)	$f_{vib}$ (Hz)	$A_{vib}$ (μm)	$\theta_{rel}$ (deg)	${LTD}_{reg}$ (LTD_sim_)	${SCR}_{reg}$ (SCR_sim_)	${S_{q}}_{reg}$ $\left({S_{q}}_{sim}\right)$	${Ssk}_{reg}$ (Ssk_sim_)	${Sku}_{reg}$ (Sku_sim_)
**Doctor Blade-** **No-Vib.**	0.01	0	0	0	- (28.9)	- (98.56)	- (20.9)	−0.716 (-)	3.962 (-)
**Vib. Doctor Blade-Opt.**	0.01	2000	5	0	21.4 (22.1)	99.54 (99.33)	15.8 (15.7)	−1.771 (−1.543)	6.766 (6.530)
	$f_{vib}$ **(Hz)**	$A_{vib}$ **(μm)**	$\theta_{tap}$ **(deg)**	$h_{bf}$ **(mm)**	${LTD}_{sim}$	${SCR}_{sim}$	${S_{q}}_{sim}$	${Ssk}_{sim}$	${Sku}_{sim}$
**Sieving-** **Opt. Sku-Ssk**	500	50	4	1.5	20.0	99.47	16.2	−0.951	5.550

**Table 9:** Trial #26 sieving data.

*TRIAL #*	f_vib_ (Hz)	A_vib_ (μm)	θ_tap_ (deg.)	h_bf_ (mm)	Q (mg/s)	m (mg)	LESR	t_lin_ (s)
***26***	**500**	**50**	**4**	**1.5**	0.239	0.452	0.788	2.025

**Table 10:** Layer quality (examined via Ssk, Sku) and the relation between linearity duration and the time for depositing standard mass of powder (0.205 mg) with the linearity mass flow. Color code: from red to green signals worst through to best performance.

*TRIAL* *#*	f_vib_ (Hz)	A_vib_ (μm)	θ_tap_ (deg.)	h_bf_ (mm)	Q (mg/s)	m (mg)	t_lin_ (s)	t_0.205_	t_lin_-t_0.205_	m-m_des_	Ssk	Sku	Ssk-Ssk_DB-No-vib_	Sku-Sku_DB-No-vib_
***1***	**200**	**20**	**1**	**0.5**	1.141	0.183	0.171	0.180	−0.009	−0.022	−0.788	4.269	−0.072	0.307
***2***	**200**	**20**	**4**	**1**	0.888	0.238	0.271	0.231	0.040	0.033	−0.786	4.583	−0.070	0.621
***3***	**200**	**20**	**7**	**1.5**	0.9	0.218	0.243	0.228	0.015	0.013	−0.794	4.571	−0.078	0.609
***4***	**200**	**35**	**1**	**1**	0.844	0.429	0.523	0.243	0.280	0.224	−0.745	4.312	−0.029	0.350
***5***	**200**	**35**	**4**	**1.5**	0.737	0.659	0.923	0.278	0.645	0.454	−0.747	4.338	−0.031	0.376
***6***	**200**	**35**	**7**	**0.5**	1.082	0.152	0.145	0.189	−0.044	−0.053	−0.732	4.293	−0.016	0.331
***7***	**200**	**50**	**1**	**1.5**	0.576	0.296	0.501	0.356	0.145	0.091	−0.682	4.372	0.034	0.410
***8***	**200**	**50**	**4**	**0.5**	0.75	0.11	0.147	0.273	−0.126	−0.095	−0.650	3.948	0.066	−0.014
***9***	**200**	**50**	**7**	**1**	0.673	0.341	0.523	0.305	0.218	0.136	−0.800	4.719	−0.084	0.757
***10***	**350**	**20**	**1**	**1**	0.771	0.427	0.578	0.266	0.312	0.222	−0.834	4.476	−0.118	0.514
***11***	**350**	**20**	**4**	**1.5**	0.584	0.664	1.183	0.351	0.832	0.459	−0.770	4.499	−0.054	0.537
***12***	**350**	**20**	**7**	**0.5**	1.075	0.146	0.14	0.191	−0.051	−0.059	−0.400	3.884	0.316	−0.078
***13***	**350**	**35**	**1**	**1.5**	0.404	0.566	1.388	0.507	0.881	0.361	−0.832	4.666	−0.116	0.704
***14***	**350**	**35**	**4**	**0.5**	0.594	0.102	0.176	0.345	−0.169	−0.103	−0.455	3.701	0.261	−0.261
***15***	**350**	**35**	**7**	**1**	0.536	0.334	0.64	0.382	0.258	0.129	−0.809	4.924	−0.093	0.962
***16***	**350**	**50**	**1**	**0.5**	0.372	0.083	0.233	0.551	−0.318	−0.122	−0.482	3.385	0.234	−0.577
***17***	**350**	**50**	**4**	**1**	0.334	0.269	0.811	0.614	0.197	0.064	−0.864	5.165	−0.148	1.203
***18***	**350**	**50**	**7**	**1.5**	0.307	0.462	1.525	0.668	0.857	0.257	−0.873	4.643	−0.157	0.681
***19***	**500**	**20**	**1**	**1.5**	0.408	0.648	1.622	0.502	1.120	0.443	−0.784	4.286	−0.068	0.324
***20***	**500**	**20**	**4**	**0.5**	0.983	0.111	0.115	0.209	−0.094	−0.094	−0.421	3.896	0.295	−0.066
***21***	**500**	**20**	**7**	**1**	0.696	0.359	0.535	0.295	0.240	0.154	−0.802	4.517	−0.086	0.555
***22***	**500**	**35**	**1**	**0.5**	0.496	0.076	0.159	0.413	−0.254	−0.129	−0.536	3.559	0.180	−0.403
***23***	**500**	**35**	**4**	**1**	0.421	0.282	0.681	0.487	0.194	0.077	−0.851	4.965	−0.135	1.003
***24***	**500**	**35**	**7**	**1.5**	0.338	0.522	1.523	0.607	0.916	0.317	−0.865	4.792	−0.149	0.830
***25***	**500**	**50**	**1**	**1**	0.278	0.265	0.969	0.737	0.232	0.06	−0.839	5.074	−0.123	1.112
***26***	**500**	**50**	**4**	**1.5**	0.239	0.452	2.025	0.858	1.167	0.247	−0.951	5.550	−0.235	1.588
***27***	**500**	**50**	**7**	**0.5**	0.347	0.065	0.205	0.591	−0.386	−0.14	−0.430	3.316	0.286	−0.646

**Table 11:** Layer quality for each trial by increasing order. Color code: from red to green signals worst through to best performance.

*TRIAL* *#*	f_vib_ (Hz)	A_vib_ (μm)	θ_tap_ (deg.)	h_bf_ (mm)	t_lin_-t_0.205_ (s)	m-m_des_ (mg)	LTD (μm)	SCR (%)	S_q_ (μm)	PD_tr_ (%)	Ssk-Ssk_DB-No-vib_	Sku-Sku_DB-No-vib_	Rating (%)
***27***	**500**	**50**	**7**	**0.5**	−0.386	−0.14	−29.6	99.2	20.3	68.7	0.286	−0.646	2.7
***16***	**350**	**50**	**1**	**0.5**	−0.318	−0.122	−22	99.3	21.4	68.4	0.234	−0.577	9.0
***12***	**350**	**20**	**7**	**0.5**	−0.051	−0.059	−19.8	99.4	19.9	67.9	0.316	−0.078	12.7
***14***	**350**	**35**	**4**	**0.5**	−0.169	−0.103	−20.5	99.3	20.5	68.3	0.261	−0.261	13.6
***20***	**500**	**20**	**4**	**0.5**	−0.094	−0.094	−19.8	99.3	20.1	67.9	0.295	−0.066	14.9
***22***	**500**	**35**	**1**	**0.5**	−0.254	−0.129	−21.8	99.3	21	68.5	0.18	−0.403	17.8
***8***	**200**	**50**	**4**	**0.5**	−0.126	−0.095	−21.4	99.3	19.2	68.1	0.066	−0.014	36.8
***7***	**200**	**50**	**1**	**1.5**	0.145	0.091	−20.6	99.4	18.1	67.5	0.034	0.41	49.2
***6***	**200**	**35**	**7**	**0.5**	−0.044	−0.053	−21.3	99.3	18.5	68.1	−0.016	0.331	52.0
***4***	**200**	**35**	**1**	**1**	0.28	0.224	−20.4	99.3	18.5	67.3	−0.029	0.35	53.6
***5***	**200**	**35**	**4**	**1.5**	0.645	0.454	−20.7	99.3	18.7	67.4	−0.031	0.376	54.4
***1***	**200**	**20**	**1**	**0.5**	−0.009	−0.022	−21	99.3	19	67.8	−0.072	0.307	56.5
***19***	**500**	**20**	**1**	**1.5**	1.12	0.443	−20.7	99.3	19	67.8	−0.068	0.324	56.6
***11***	**350**	**20**	**4**	**1.5**	0.832	0.459	−19.9	99.4	18.1	67.4	−0.054	0.537	60.1
***21***	**500**	**20**	**7**	**1**	0.24	0.154	−20.3	99.3	18.1	67.6	−0.086	0.555	63.4
***2***	**200**	**20**	**4**	**1**	0.04	0.033	−20.2	99.3	18.2	67.5	−0.07	0.621	63.4
***3***	**200**	**20**	**7**	**1.5**	0.015	0.013	−20.5	99.3	18.1	67.4	−0.078	0.609	63.8
***10***	**350**	**20**	**1**	**1**	0.312	0.222	−20.4	99.4	18.4	67.6	−0.118	0.514	65.3
***9***	**200**	**50**	**7**	**1**	0.218	0.136	−20.3	99.4	17.4	67.4	−0.084	0.757	67.7
***13***	**350**	**35**	**1**	**1.5**	0.881	0.361	−20.2	99.3	17.9	67.4	−0.116	0.704	69.4
***18***	**350**	**50**	**7**	**1.5**	0.857	0.257	−20.5	99.3	18	67.4	−0.157	0.681	72.6
***15***	**350**	**35**	**7**	**1**	0.258	0.129	−20.6	99.4	17	67.5	−0.093	0.962	73.1
***24***	**500**	**35**	**7**	**1.5**	0.916	0.317	−20.5	99.4	17.4	67.4	−0.149	0.83	75.2
***23***	**500**	**35**	**4**	**1**	0.194	0.077	−19.9	99.4	17	66.9	−0.135	1.003	77.8
***25***	**500**	**50**	**1**	**1**	0.232	0.06	−20	99.5	16.2	67.0	−0.123	1.112	79.2
***17***	**350**	**50**	**4**	**1**	0.197	0.064	−19.9	99.4	16.4	67.0	−0.148	1.203	83.5
***26***	**500**	**50**	**4**	**1.5**	1.167	0.247	−20	99.4	16.2	67.1	−0.235	1.588	100.0
***Average***					-	-	−20.8	99.3	18.5	67.6	−0.007	0.434	-
***Standard Deviation***					-	-	1.8	0.1	1.4	0.5	-	-	-
***Standard Deviation (%)***					-	-	8.8	0.1	7.6	0.7	-	-	-

## Data Availability

The data presented in this study are not available on request from the corresponding authors because they are part of an ongoing study.

## Appendix A. ANOVA Results

**Table A1:** ANOVA for total mass sieved in linearity.

Source	DF	Seq SS	Contribution	Adj SS	Adj MS	F-Value	*p*-Value
**Regression**	7	0.779953	84.58%	0.779953	0.111422	14.89	0.000
**f_vib_ (Hz)**	1	0.001318	0.14%	0.000243	0.000243	0.03	0.859
**A_vib_ (μm)**	1	0.023544	2.55%	0.000646	0.000646	0.09	0.772
**θ_tap_ (deg.)**	1	0.007771	0.84%	0.038923	0.038923	5.20	0.034
**h_bf_ (mm)**	1	0.664705	72.09%	0.017935	0.017935	2.40	0.138
**f_vib_ (Hz)*A_vib_ (μm)**	1	0.016428	1.78%	0.016428	0.016428	2.20	0.155
**f_vib_ (Hz)*h_bf_ (mm)**	1	0.034347	3.72%	0.018180	0.018180	2.43	0.136
**A_vib_ (μm)*θ_tap_ (deg.)**	1	0.031840	3.45%	0.031840	0.031840	4.26	0.053
**Error**	19	0.142149	15.42%	0.142149	0.007482		
**Total**	26	0.922102	100.00%				

**Table A2:** ANOVA for mass flow during linearity.

Source	DF	Seq SS	Contribution	Adj SS	Adj MS	F-Value	*p*-Value
**Regression**	7	176.532	92.86%	176.532	0.252189	35.32	0.000
**f_vib_ (Hz)**	1	0.63657	33.49%	0.03887	0.038869	5.44	0.031
**A_vib_ (μm)**	1	0.70805	37.25%	0.11374	0.113745	15.93	0.001
**θ_tap_ (deg.)**	1	0.02449	1.29%	0.01715	0.017145	2.40	0.138
**h_bf_ (mm)**	1	0.30602	16.10%	0.12230	0.122304	17.13	0.001
**f_vib_ (Hz)*A_vib_ (μm)**	1	0.00715	0.38%	0.00386	0.003864	0.54	0.471
**A_vib_ (μm)*h_bf_ (mm)**	1	0.07680	4.04%	0.07680	0.076800	10.75	0.004
**θ_tap_ (deg.)*h_bf_ (mm)**	1	0.00623	0.33%	0.00623	0.006230	0.87	0.362
**Error**	19	0.13568	7.14%	0.13568	0.007141		
**Total**	26	190.100	100.00%				

**Table A3:** ANOVA for total duration of linear sieving stage.

Source	DF	Seq SS	Contribution	Adj SS	Adj MS	F-Value	*p*-Value
**Regression**	7	747.360	94.69%	747.360	106.766	48.40	0.000
**f_vib_ (Hz)**	1	106.921	13.55%	0.20059	0.20059	9.09	0.007
**A_vib_ (μm)**	1	0.24059	3.05%	0.04630	0.04630	2.10	0.164
**θ_tap_ (deg.)**	1	0.02457	0.31%	0.13670	0.13670	6.20	0.022
**h_bf_ (mm)**	1	495.285	62.75%	0.06750	0.06750	3.06	0.096
**f_vib_ (Hz)*h_bf_ (mm)**	1	101.326	12.84%	0.79268	0.79268	35.93	0.000
**A_vib_ (μm)*θ_tap_ (deg.)**	1	0.11375	1.44%	0.11375	0.11375	5.16	0.035
**A_vib_ (μm)*h_bf_ (mm)**	1	0.05936	0.75%	0.05936	0.05936	2.69	0.117
**Error**	19	0.41913	5.31%	0.41913	0.02206		
**Total**	26	789.272	100.00%				
